# TcellInflamedDetector: an R package to distinguish T cell inflamed tumor types from non–T cell inflamed tumor types

**DOI:** 10.5808/gi.22005

**Published:** 2022-03-31

**Authors:** San-Duk Yang, Hyun-Seok Park

**Affiliations:** 1 School of Integrated Software and Design, Kyung Hee Cyber University, Seoul 02447, Korea; 2 Bioinformatics Laboratory, ELTEC College of Engineering, Ewha Womans University, Seoul 03760, Korea; 3 Center for Convergence Research of Advanced Technologies, Ewha Womans University, Seoul 03760, Korea

**Keywords:** gene expression, immune checkpoint inhibitors, immunotherapy, prognosis, RNA-seq, software

## Abstract

A major issue in the use of immune checkpoint inhibitors is their lack of efficacy in many patients. Previous studies have reported that the T cell inflamed signature can help predict the response to immunotherapy. Thus, many studies have investigated mechanisms of immunotherapy resistance by defining the tumor microenvironment based on T cell inflamed and non–T cell inflamed subsets. Although methods of calculating T cell inflamed subsets have been developed, valid screening tools for distinguishing T cell inflamed from non–T cell inflamed subsets using gene expression data are still needed, since general researchers who are unfamiliar with the details of the equations can experience difficulties using extant scoring formulas to conduct analyses. Thus, we introduce TcellInflamedDetector, an R package for distinguishing T cell inflamed from non–T cell inflamed samples using cancer gene expression data via bulk RNA sequencing.

## Introduction

Cancer cells express programmed death ligand 1 as a signal related to T cell unresponsiveness. Immunotherapies targeting immune checkpoints (e.g., anti–cytotoxic T lymphocyte associated antigen-4 and anti–programmed death-1 antibodies) are a standard component of care for patients with advanced cancers. Immune checkpoint inhibitors (ICIs) have led to improvements in the survival rate, but only a subset of patients respond to ICIs. Recent studies have reported that the efficacy of ICIs in cancer patients is determined by the T cell inflamed tumor microenvironment [1-3]. The molecular mechanisms of resistance have not yet been elucidated in detail. Nevertheless, previous studies have reported scoring methods for distinguishing non–T cell inflamed from T cell inflamed tumors based on gene expression data [4,5].

Unfortunately, general researchers who are unfamiliar with the detailed calculations involved in the equations can experience difficulties using these scoring formulas to conduct analyses. For this reason, we recently developed TcellInflamedDetector, an R package that predicts T cell inflamed tumors when given RNA-sequencing expression data. This package will be beneficial to optimize the selection of patients predicted to benefit from ICIs. TcellInflamedDetector implements the equation developed by Spranger et al. [5] to differentiate non–T cell inflamed and T cell inflamed tumor subtypes.

## Input Data and Processing

**Fig. 1.**
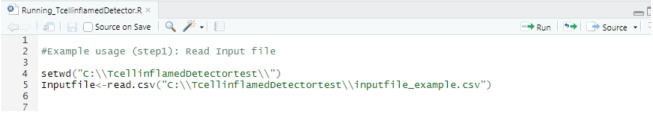
An exemplary code for extraction of T cell effector gene subset.

As shown in Fig. 1, TcellInflamedDetector requires RNA-sequencing count input data with genes and sample identifiers. Users follow the steps for data processing that are summarized in the TcellInflamedDetector manual on GitHub [6]. The input CSV file is RNA sequencing log count per million (CPM) data. The count matrix file is converted by EdgeR aveLogCPM() and the calcNormFactor function using the trimmed mean of the m-values method. Users can extract previously established gene signatures indicative of a T cell inflamed tumor microenvironment, which include the cytotoxic T lymphocyte (CTL) signature genes *CD8A, CD8B, GZMA, GZMB*, and *PRF1* using R code [7-10]. The established gene signatures were referenced with the Gajewski T cell-inflamed signature, interferon-gamma related signature, T cell effector signature, and immune cytolytic activity signature [4,5].

## Estimating T Cell Inflamed and Non–T Cell Inflamed Samples

**Fig. 2.**
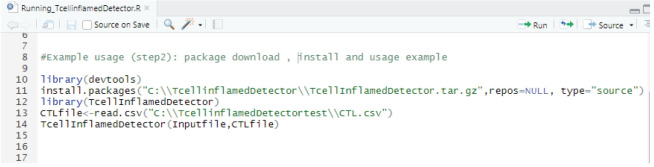
An exemplary usage of R code.

As shown in Fig. 2, gene expression values were converted to a score *S_i_* = *µ_i_* ± *β_i_*σ*_i_* (i = 1, 2, … n), where µ and σ represent the mean and standard deviation (SD) of the i^th^ gene’s expression across all samples, n is the total number of genes, *β* represents the distance between the i^th^ gene’s expression in a sample and the mean in units of the SD (equivalent to a z-score). The threshold for non–T cell inflamed and T cell inflamed tumors was *β_0_* = 0.1. The algorithm is described in detail below:

If the z-score value *β_i_* is greater than the threshold (*β_0_* = 0.1), then +1 is assigned. Otherwise, if the z-score value *β_i_* is less than the threshold (*β_0_* = 0.1), then ‒1 is assigned. If the sum of the column of genes with assigned values is greater than half of the number of CTL genes, then the output is a classification of “T cell inflamed.” If the sum of a column of genes with assigned values is less than half of the number of CTL genes, then the classification is “non–T cell inflamed.” Otherwise, the sample is classified as “intermediate.”

Users of the R package can obtain results in the format of a .csv file that contains data on the classification of samples as T cell inflamed, non–T cell inflamed, and intermediate. If users want to modify the CTL gene list when running the R package, they do not have to modify the complex R code. Instead, they can simply revise the gene list contained in the CTL.csv file.

## Output

**Fig. 3.**
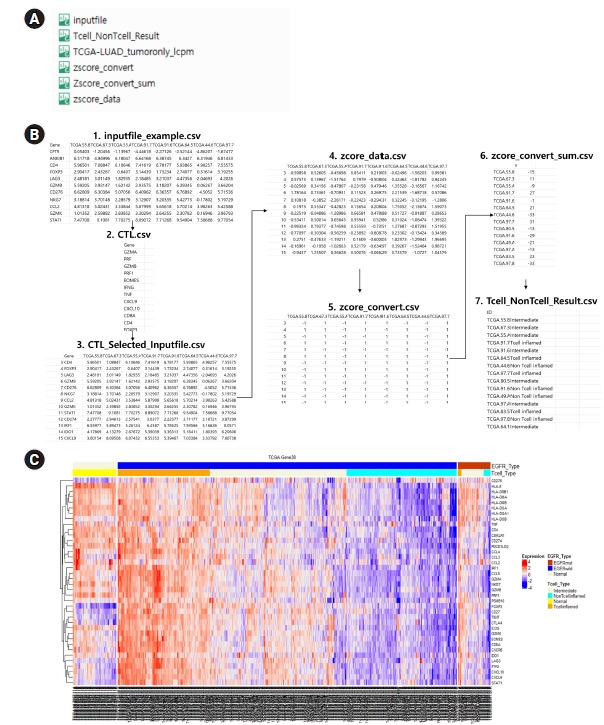
(A) An input file and generated output files. (B) Processing diagram for T cell inflamed function prediction. (C) T cell inflamed annotation of Heatmap expression in The Cancer Genome Atlas (TCGA) lung adenocarcinoma (LUAD) samples.

Five output formats are available: CTL_Selected_Inputfile.csv, Tcell_NonTcell_Result.csv, zscore_convert.csv, Zscore_convert_sum.csv, and zscore_data.csv. Fig. 3 presents examples of the prediction results of T cell inflamed, intermediate, and non–T cell inflamed groups. Users can check the expression patterns of specific genes through a heatmap. We also confirmed that T cell inflamed samples showed high expression of T cell effector gene signatures [10].

Finally, we conducted a test to demonstrate our tool’s flexibility; we tested it on The Cancer Genome Atlas (TCGA) lung adenocarcinoma RNA-sequencing dataset available through the TCGA Research Network [11]. Each sample was labeled according to the TCGA barcode, which contained gene names. Our package successfully selected subsets of gene expression data from the raw count data. Thus, TcellInflamedDetector can be beneficial for future cancer immunotherapy vaccine developers and researchers.
